# Memantine treatment in individuals with GRIN gain‐of‐function variants is associated with improvements in behavior, development, and seizure frequency

**DOI:** 10.1002/epi.70090

**Published:** 2026-01-05

**Authors:** Maike Karnstedt, Riley E. Perszyk, Scott J. Myers, Ellington McDaniels, Marta Somorai, Ingo Borggraefe, Danielle C. M. Veenma, An‐Sofie Schoonjans, Pasquale Striano, Tadeu A. Fantaneanu, Steffen Syrbe, Kristen Park, Wenjuan Chen, Hongjie Yuan, Stephen F. Traynelis, Timothy A. Benke, Johannes R. Lemke, Ilona Krey

**Affiliations:** ^1^ Institute of Human Genetics University of Leipzig Medical Center Leipzig Germany; ^2^ Department of Pharmacology and Chemical Biology Emory University School of Medicine Atlanta Georgia USA; ^3^ Center for Functional Evaluation of Rare Variants Emory University School of Medicine Atlanta Georgia USA; ^4^ Center for Rare Developmental Disorders, Technical University of Munich, kbo Children's Center Munich Munich Germany; ^5^ Division of Pediatric Neurology, Developmental Medicine, and Social Pediatrics, Department of Pediatrics University Hospital Munich Munich Germany; ^6^ Department of Pediatrics Erasmus MC Sophia Children's Hospital Rotterdam the Netherlands; ^7^ Department of Pediatric Neurology Antwerp University Hospital Edegem Belgium; ^8^ Department of Neurosciences, Rehabilitation, Ophthalmology, Genetics, Maternal and Child Health University of Genoa Genoa Italy; ^9^ IRCCS Istituto Giannina Gaslini Genoa Italy; ^10^ Department of Medicine, Ottawa Hospital, Ottawa Hospital Research Institute University of Ottawa Ottawa Ontario Canada; ^11^ Division of Pediatric Epileptology, Center for Pediatrics and Adolescent Medicine University Hospital Heidelberg Heidelberg Germany; ^12^ Departments of Pediatrics, Pharmacology, Neurology, and Otolaryngology, School of Medicine and Children's Hospital Colorado University of Colorado Boulder Colorado USA; ^13^ Center for Neurodegenerative Disease Emory University School of Medicine Atlanta Georgia USA; ^14^ Center for Rare Diseases University of Leipzig Medical Center Leipzig Germany; ^15^ Present address: Department of Psychiatry, Sir Run Run Shaw Hospital Zhejiang University School of Medicine Hangzhou China

**Keywords:** developmental and epileptic encephalopathy, epilepsy, precision medicine, retrospective observational case series, targeted treatment

## Abstract

**Objective:**

GRIN‐related disorders due to pathogenic variants in *GRIN1*, *GRIN2A*, *GRIN2B*, or *GRIN2D* genes are associated with altered N‐methyl‐D‐aspartate receptor (NMDAR) function. Functional changes include gain (GoF) and loss of receptor function (LoF). Clinical reports describing the use of the NMDAR blocker memantine in GRIN‐related disorders show a diverse and inconsistent spectrum of treatment responses.

**Methods:**

To evaluate clinical responses to memantine, we collected retrospective data on 34 individuals with GRIN variants, including 20 unpublished and 14 published cases. Variants were reclassified following American College of Medical Genetics and Genomics guidelines, and six in vitro functional assays were used to assess receptor function. We compared individuals with pathogenic GoF versus LoF in terms of associated clinical improvements, memantine sensitivity, and variant localization within the gene.

**Results:**

In 19 of the 34 variants, a pathogenic likely or possible GoF of the receptor was detected. Fourteen of 19 individuals (74%) benefited from memantine, comprising improvements in behavior (71%), development (50%), and seizure frequency (39%). Individuals with either LoF or a functionally indeterminate or no effect GRIN variant (15/34 individuals) showed significantly less benefit from memantine treatment but nevertheless rare adverse events (3/15). An increased distance of the variant from the memantine binding site was associated with a clinical benefit.

**Significance:**

Our retrospective observational study outlines the importance of correct classification of GRIN variants with regard to pathogenicity and functional consequence prior to applying memantine or other precision medicine approaches in clinical trials. Furthermore, the distance from a GoF variant to the memantine binding site correlated with a positive treatment response and may, at least in part, explain different degrees of therapeutic benefit.

**Key points**

- We provide the first replication showing that memantine benefits only individuals with GRIN gain‐of‐function variants.
- Most prior GRIN case reports ignored variant function, leading to inappropriate memantine use and obscuring true clinical benefit.
- We could identify that an increased distance to the memantine binding site leads to clinical benefits in carriers of gain‐of‐function variants.
- Given limited treatment options for GRIN disorders, our findings support memantine as a promising precision medicine approach.

## 1 INTRODUCTION

Glutamate receptors mediate rapid excitatory signaling in the central nervous system and regulate diverse functions across the brain, spinal cord, retina, and peripheral nervous system. They are thought to play a key role in various neurological processes, making them a focus of extensive research.^1^, ^2^ One subtype is ionotropic glutamate receptors, including the N‐methyl‐D‐aspartate receptor (NMDAR). Ionotropic glutamate receptors play an essential role in normal brain function, and their dysfunction is associated with various diseases.^2^ NMDAR activation produces inward currents with high permeability to Ca^2+^ when voltage‐dependent Mg^2+^ block is relieved by depolarization. The tetrameric structure of NMDAR consists of two glycine‐binding GluN1 and two glutamate‐binding GluN2 subunits, and NMDAR activation requires the concurrent binding of the coagonists glycine (or D‐serine) and glutamate. The GluN1 subunit is encoded by *GRIN1*, whereas the GluN2 subunit is derived from *GRIN2A‐D*.^2^

In this study, we focused on variants in the clinically relevant GRIN genes associated with neurological disorders such as developmental delay, intellectual disability, epilepsy, and autism, summarized as developmental and epileptic encephalopathy (DEE).^3^ The functional consequences of a respective GRIN variant are hypothesized to guide more precise treatments. At present, the therapeutic rationale and evidence suggest missense variants with a loss‐of‐function (LoF) effect may respond to coagonistic treatment that could elevate overall NMDAR activation,^4^, ^5^ whereas gain‐of‐function (GoF) variants may benefit from treatment with inhibitors that block the NMDAR, thereby reducing overactivation.

Memantine is an NMDAR blocker binding directly in the channel pore.^6^, ^7^ Memantine is frequently used to treat Alzheimer disease, but has also been associated with therapeutic improvements in a limited number of individuals with GRIN‐related disorders, including reduced seizure frequency and improved behavior.^3^, ^8^, ^9^, ^10^ However, a further evaluation of these individuals revealed that several GRIN variants were not classified correctly and/or were lacking functional GoF verification.

This retrospective observational study examined the genetic data, clinical description, and treatment response in 34 individuals with GRIN‐related disorders. We examined which variants respond to memantine treatment with regard to spectrum and extent of therapeutic improvements. We also investigated the impact of the localization of the variant on the therapeutic outcome.

## 2 MATERIALS AND METHODS

### 2.1 Patient cohort

We collected a cohort of 34 individuals, comprising 14 previously published cases^8^, ^9^, ^10^, ^11^, ^12^, ^13^, ^14^, ^15^, ^16^ and an additional 20 novel cases from the GRI Registry originating from the United States (*n* = 8), Germany (*n* = 5), the Netherlands (*n* = 3), Italy (*n* = 3), and the UK (*n* = 1). In all registered cases, it was stated that treatment with memantine was administered.

We reclassified all variants according to the current guidelines of the American College of Medical Genetics and Genomics (ACMG).^17^

Among individuals with pathogenic or likely pathogenic GRIN variants who received memantine, we documented details of memantine application and collected data on potential treatment responses.

Standard treatment with memantine was .5 mg/kg/day. The dose was increased for a few cases up to 2 (#16) or even 4 mg/kg/day (#15). Treatment duration ranged from a couple of days to several months. The longest period of time recorded was 22 months. For seven cases, the duration of treatment was not specified. The identity of the supplier of memantine is not disclosed.

Treatment response was subjectively assessed through the clinician's personal evaluation and feedback from parents and caregivers (see Table S1). Efficacy was rated in four different categories that included behavior, cognitive/global development, electroencephalography (EEG), and seizure frequency. These clinical categories rely on previous precision medicine approaches in GRIN‐related disorders.^4^, ^5^

Different standardized neurodevelopmental tests were performed in four cases, including the Bayley Scales of Infant Development, Third Edition^18^ in two individuals (#4 and #19) and the Münchener Funktionelle Entwicklungsdiagnostik 1–4^19^ in two individuals (#1 and #5).

Thus, our report represents an unblinded, retrospective, observational multicenter analysis, not meeting the standards of a randomized controlled trial. This retrospective data collection has been approved by the ethics committee of the University of Leipzig (224/16‐ek and 379/21‐ek) and the University of Colorado (COMIRB 16‐1520). Informed and voluntary consent according to the European General Data Protection Regulation and the Declaration of Helsinki has been obtained from each subject or legal guardian prior to recruitment and investigation.

### 2.2 Analysis of variant effects on NMDAR function

We employed five electrophysiological and one biochemical in vitro assay to determine the functional consequence of each variant, as previously described.^20^ Furthermore, all variants have been tested for memantine IC_50_ values. Briefly, the patient‐specific variants were introduced into a wild‐type (WT) human cDNA encoding GluN1, GluN2A, GluN2B, or GluN2D (GenBank: NM_007327, NM_000833, NM_000834, and NM_000836) and the variant NMDARs expressed in *Xenopus laevis* oocytes (Glu, Gly, Mg^2+^, P_OPEN_) and HEK293 cells (*Tau*_W_ and surface expression). See Supplemental Material and Methods for a complete description of these assay conditions.

Variants were classified as GoF, LoF, or indeterminate and are reported in Tables S1 according to threshold‐driven determinations as per previously published criteria (see also Tables S3A and S3B).^20^ Threshold combinations were taken into account resulting in “likely GoF” if one or more changes demonstrate “high confidence” without any indication of LoF across the assays. A “possible GoF” was assigned when at least two assays reach a “moderate confidence” level. “Likely GoF” and “possible GoF” were considered to be indication for treatment with the NMDAR blocker memantine. In cases where the six assays reveal both high or moderate confidence for GoF as well as high or moderate confidence for LoF, a variant was considered “indeterminate” if the net estimated change in ion channel function did not meet criteria to suggest promotion to GoF or LoF.^20^ If no high‐confidence alteration to any functional parameter was detectable and fewer than two moderate changes were observed, a variant was defined as having “no effect.” Together with clear LoF and indeterminate variants, this classification should have prompted caution regarding the use of this medication.

## 3 RESULTS

### 3.1 Variant classification

Our cohort of 34 individuals comprised 29 different GRIN variants (Table 1). After applying current ACMG criteria, 24 of these 29 variants were classified as (likely) pathogenic, comprising 10 variants in *GRIN1* (12 individuals), three variants in *GRIN2A* (three individuals), 10 variants in *GRIN2B* (11 individuals), and one variant in *GRIN2D* (three individuals). Five nonpathogenic variants were classified as variants of unknown significance (VUSs; #8, #19, #29,^13^ and #31^15^) or likely benign (#28^14^), refuting a diagnosis of a GRIN‐related disorder.

**TABLE 1:** Overview of all 34 individuals treated with memantine sorted by GoF (Groups 1–3, upper part of the table) and LoF, indeterminate, and no effect variants (Group 4, lower part of the table) using the criteria described by Myers et al.^20^

Individual #	Age, years	Sex	Gene	Variant c.	Variant p.	Variant type	Location	Origin	ACMG conclusion	Functional consequences	Clinical and variant; data source (PMID)	WT memantine IC_50_, mmol·L^−1^ [95% CI] (*n*)^a^	Variant memantine IC_50_, mmol·L^−1^ [95% CI] (*n*)^a^	Memantine data source (PMID)	Group by clinical response to memantine
14	17	F	*GRIN1*	c.2116A>G	p.(Met706Val)	Missense	S2	De novo	P	Possible GoF	Registry	4.9 [4.2–5.7] (27)	4.1 [3.2–5.1] (16)	This study	Group 1: GoF improved
27	4	M	*GRIN1*	c.1923G>A	p.(Met641Ile)	Missense	M3	De novo	P	Possible GoF	34227748	5.1 [4.3–5.9] (61)	1.3 [.92–1.6] (23)^b^	34227748	
20	11	M	*GRIN2A*	c.2434C>A	p.(Leu812Met)	Missense	Pre‐M4	De novo	P	Likely GoF	24839611, 39535073	4.6 [3.6–5.6] (15)	12 [10–14] (10)^b^	24839611	
1	9	M	*GRIN2B*	c.1621A>G	p.(Ser541Gly)	Missense	S1‐M1	De novo	P	Possible GoF	Registry	1.0 [.91–1.1] (15)	1.4 [1.3–1.6] (8)^b^	This study	
5	9	F	*GRIN2B*	c.2087G>A	p.(Arg696His)	Missense	S2	De novo	P	Likely GoF	Registry	1.8 [1.4–2.2] (13)	2.1 [1.5–2.7] (10)	27839871	
21	8	n.a.	*GRIN2B*	c.1832G>T	p.(Gly611Val)	Missense	M2	De novo	P	Possible GoF	28377535, 37369021	1.5 [1.1–1.8] (18)	1.9 [1.3–2.6] (10)	37649269	
24	8	n.a.	*GRIN2B*	c.2453 T>C	p.(Met818Thr)	Missense	M4	De novo	P	Likely GoF	28377535, 39535073	1.7 [1.3–2.1] (26)	3.0 [2.0–4.0] (9)	28377535	
26	3	M	*GRIN2B*	c.2453A>T	p.(Met818Leu)	Missense	M4	De novo	P	Possible GoF	34844267	1.0 [.91–1.1] (15)	2.3 [1.9–2.7] (11)^b^	This study	
33	11	F	*GRIN2D*	c.1999G>A	p.(Val667Ile)	Missense	M3	De novo	P	Possible GoF	27616483	.57 [.49–.65] (10)	4.0 [2.2–5.8] (9)^b^	27616483	
34	11	M	*GRIN2D*	c.1999G>A	p.(Val667Ile)	Missense	M3	De novo	P	Possible GoF	Registry	.57 [.49–.65] (10)	4.0 [2.2–5.8] (9)^b^	27616483	
11	8	F	*GRIN1*	c.1921A>G	p.(Met641Val)	Missense	M3	De novo	P	Possible GoF	Registry	4.9 [4.2–5.7] (27)	1.8 [1.4–2.3] (11)^b^	This study	Group 2: GoF improved, followed by habituation
7	11	F	*GRIN2A*	c.1936A>G	p.(Thr646Ala)	Missense	M3	De novo	P	Possible GoF	Registry	4.8 [4.1–5.7] (56)	>100 (12)^b^	38538865	
3	18	F	*GRIN2B*	c.1832G>T	p.(Gly611Val)	Missense	M2	De novo	P	Possible GoF	Registry	1.5 [1.1–1.8] (18)	1.9 [1.3–2.6] (10)	37649269	
22	8	n.a.	*GRIN2B*	c.1844A>T	p.(Asn615Ile)	Missense	M2	De novo	P	Possible GoF	28377535, 37369021	1.5 [1.1–1.8] (18)	.35 [.3–.4] (11)^b^	37649269	
10	8	M	*GRIN1*	c.1923G>A	p.(Met641Ile)	Missense	M3	De novo	P	Possible GoF	Registry	5.1 [4.3–5.9] (61)	1.3 [.92–1.6] (23)^b^	34227748	Group 3: GoF not improved
13	22	F	*GRIN1*	c.2116A>G	p.(Met706Val)	Missense	S2	De novo	P	Possible GoF	Registry	4.9 [4.2–5.7] (27)	4.1 [3.2–5.1] (16)	This study	
6	12	M	*GRIN2A*	c.1930A>G	p.(Ser644Gly)	Missense	M3	De novo	P	Possible GoF	Registry	4.8 [4.1–5.7] (56)	11 [7.3–15] (15)^b^	38538865	
25	7	F	*GRIN2B*	c.1928 T>C	p.(Leu643Pro)	Missense	M3	De novo	P	Possible GoF	30151416	1.0 [.91–1.1] (15)	44 [35–56] (8)^b^	This study	
32	17	F	*GRIN2D*	c.1999G>A	p.(Val667Ile)	Missense	M3	De novo	P	Possible GoF	27616483	.57 [.49–.65] (10)	4.0 [2.2–5.8] (9)^b^	27616483	
9	22	M	*GRIN1*	c.1852G>A	p.(Gly618Ser)	Missense	M2	De novo	P	Likely LoF	Registry	4.9 [4.2–5.7] (27)	4.6 [2.7–7.9] (8)	This study	Group 4: LoF/indeterminate/no effect/VUS
12	19	F	*GRIN1*	c.1954G>A	p.(Ala652Thr)	Missense	M2	De novo	P	Likely LoF	Registry	5.1 [4.4–5.9] (18)	2.4 [1.5–3.8] (6)^b^	This study	
15	22	F	*GRIN1*	c.2443G>T	p.(Gly815Trp)	Missense	M4	De novo	P	Possible LoF	Registry	4.9 [4.2–5.7] (27)	1.2 [1.1–1.3] (8)^b^	This study	
16	8	M	*GRIN1*	c.2443G>A	p.(Gly815Arg)	Missense	M4	De novo	P	Possible LoF	Registry	4.9 [4.2–5.7] (27)	.95 [.81–1.1] (8)^b^	This study	
17	15	M	*GRIN1*	c.2479G>A	p.(Gly827Arg)	Missense	S2	De novo	P	Likely LoF	Registry	n.a.	n.a.	‐	
18	18	M	*GRIN1*	c.2531G>C	p.(Arg844Pro)	Missense	pCTD	De novo	P	No effect	Registry	4.9 [4.2–5.7] (27)	8.3 [6.1–11] (9)^b^	This study	
30	n.a.	n.a.	*GRIN1*	c.1679_1681dup	p.(Ser560dup)	Duplication	M1	Mat inherited	P	Likely LoF	36256600, 37000222	n.a.	n.a.	‐	
31	n.a.	n.a.	*GRIN1*	c.3135_3149del15	p.(Lys1045_Ser1050delinsAsn)	Delins	CTD	Mat inherited	VUS	‐	36256600	n.a.	n.a.	‐	
8	16	F	*GRIN2A*	c.3596delC	p.(Pro1199Argfs*32)	Frameshift	CTD	De novo	VUS	Null	Registry	n.a.	n.a.	‐	
28	8	M	*GRIN2A*	c.1083G>A	p.(Leu361=)	Synonymous	ATD	Mat inherited	LB	‐	32765929	n.a.	n.a.	‐	
29	13	F	*GRIN2A*	c.2888 T>C	p.(Leu963Pro)	Missense	CTD	Mat inherited	VUS	‐	34074563	n.a.	n.a.	‐	
2	11	F	*GRIN2B*	c.1664G>T	p.(Ser555Ile)	Missense	S1‐M1	De novo	P	Likely LoF	Registry	n.a.	n.a.	‐	
4	5	M	*GRIN2B*	c.1971G>C	p.(Glu657Asp)	Missense	M3‐S2	Unknown	LP	Indeterminant	Registry	1.0 [.91–1.1] (15)	.78 [.63–.89] (8)^b^	This study	
23	8	n.a.	*GRIN2B*	c.1853 T>G	p.(Val618Gly)	Missense	M2	De novo	P	Indeterminant	28377535, 31429998	1.5 [1.1–1.8] (18)	19 [16–21] (16)^b^	28377535	
19	10	M	*GRIN2D*	c.3812C>T	p.(Ser1271Leu)	Missense	CTD	De novo	VUS	Possible LoF	Registry	.60 [.57–.63] (10)	.66 [.62–.71] (11)	This study	

*Note*: Memantine sensitivity of 27 missense variants compared to the WT variant were generated as described in Materials and Methods for this study or reproduced from the indicated papers and included here to facilitate comparison.

Abbreviations: ACMG, American College of Medical Genetics and Genomics; ATD, Amino‐terminal domain; CI, confidence interval; CTD, carboxyl‐terminal domain; Delins, deletion–insertion; F, female; GoF, gain of function; LB, likely benign; LoF, loss of function; LP, likely pathogenic; LTD (composed of S1 and S2), ligand binding domain; M, male; Mat, maternally; M1‐M4, transmembrane domains; n.a., not available; P, pathogenic; PMID, PubMed Identifier; VUS, variant of unknown significance; WT, wild type.

^a^ Data shown are the mean IC_50_ value with 95% CIs determined from the log(IC_50_) or log(EC_50_) values.

^b^ 95% CIs are nonoverlapping with WT GluN1/GluN2A‐ or GluN1/GluN2B‐containing N‐methyl‐D‐aspartate receptors, which corresponds to *p* < .01.

### 3.2 GoF versus LoF classification

Among the 24 (likely) pathogenic *GRIN* variants, 14 were classified as likely or possibly GoF of the NMDAR (accounting for 19 individuals, Groups 1–3). Group 1 comprises the individuals with improvement during memantine treatment, Group 2 those with initial improvements followed by habituation, and Group 3 those with GoF without improvements. By contrast, eight showed likely or possibly LoF of NMDAR function, accounting for eight individuals. We assigned the one case with a null variant (#8) to Group 4, due to the underlying pathomechanism of haploinsufficiency. The same assignment applies to Individual #18 with a variant leading to no effect as functional consequence, as well as Individuals #4 and #23, considered to have indeterminant variants (Table 1).

We also functionally investigated the *GRIN2D* de novo VUS c.3812C>T, p.(Ser1271Leu) of Individual #19, revealing likely LoF. This did not change the variant's classification as VUS. Thus, it remained unclear regarding disease association and was excluded from further genotype–phenotype analyses.

Through functional classification, we were able to determine a “possible GoF” for 11 variants (accounting for 16 individuals) and a “likely GoF” for three variants. The exact values of the respective assays are listed in Tables S1 and S4.

We investigated six different functional parameters as well as memantine sensitivity (measured as memantine IC_50_) to determine a variant's functionality. We identified three groups of GoF cases: 10 individuals with a positive effect during treatment with memantine (Group 1), four cases with initial improvement that dissipated over time (Group 2), and five individuals without positive or with negative effect (Group 3). The six functional parameters of Groups 1, 2, and 3, in particular differences between Group 1 and 3, did not show remarkable differences.

### 3.3 Functional characterization: Memantine sensitivity

**FIGURE 1:**
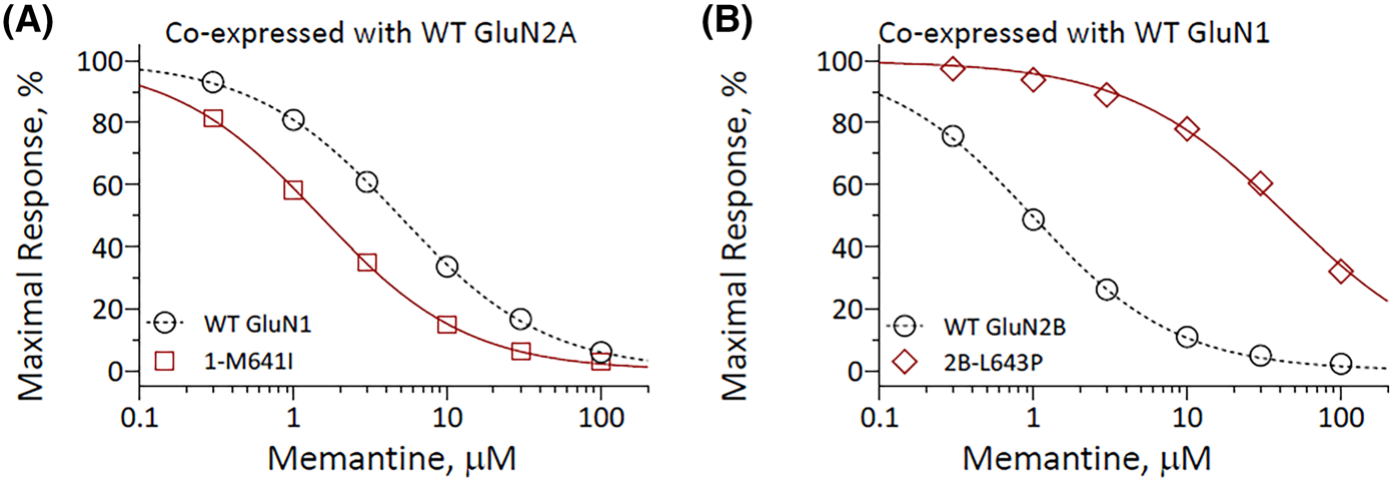
GRIN variants influence memantine sensitivity. Composite concentration–response curves of memantine were evaluated by TEVC recordings (holding potential (V_HOLD_) = −40 mV) of Xenopus oocytes expressing *GRIN2A* (A) and *GRIN1* (B) variants in the presence of maximally effective concentrations of agonists (100 μmol·L^−1^ glutamate and glycine). Data are expressed as mean ± SEM (standard error of the mean). A shows one variant of Group 1 (gain of function [GoF] with clinical improvements), B shows one variant of Group 3 (GoF without clinical improvements). WT, wild type.

We also assessed the effects individual variants had on memantine EC_50_ values of the different GoF groups (Group 1 GoF with clinical improvements [Figure 1A] vs. Group 3 GoF without clinical improvements [Figure 1B], to determine the memantine sensitivity [see also Table 1]).

Two GoF variants (*GRIN1* p.[Met706Val] and p.[Met641Ile], Individuals #14 and #27) of Group 1 were more sensitive to memantine compared to minimal block of WT receptors, which appear as a leftward shift in IC_50_, (Figure 1A). Three of five individuals of Group 3 had variants that show reduced sensitivity to memantine (i.e., a rightward shift in IC_50_), such as *GRIN2B* p.(Leu643Pro) in Individual #25 (Figure 1B). Two variants are more sensitive to memantine compared to the modest block observed for WT receptors (*GRIN1* p.[Met641Ile] and p.[Met706Val], Individuals #10 and #13).

### 3.4 Memantine‐related clinical improvements in individuals with GoF variants

The standard treatment dose of memantine was .5 mg/kg/day. Two individuals received up to 2 (#16) or even 4 mg/kg/day (#15). Memantine was taken over a period of 4 days to 22 months. Overall, memantine appeared generally well tolerated by these individuals, except for three individuals (#10, #25, and #32) who experienced increased seizure frequency. The median age of individuals was 9 years, with equally represented genders and an average treatment duration of 7 months. All 19 individuals with pathogenic GoF variants in any GRIN gene were assessed for memantine response, particularly regarding behavior, epilepsy, and development. For detailed phenotypic information, see Table S1.

**FIGURE 2:**
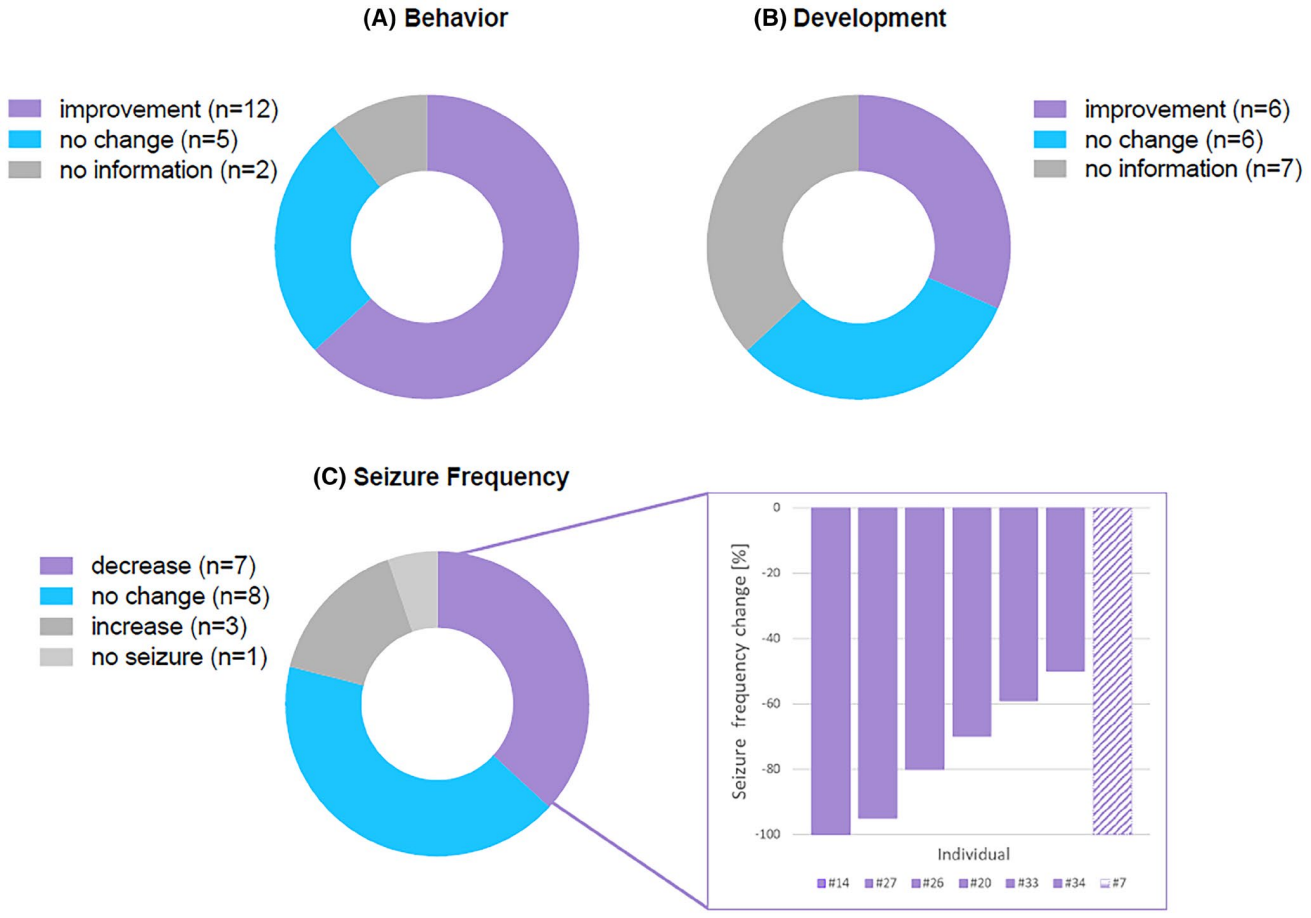
Evaluation of 19 individuals with gain‐of‐function variants in *GRIN1*, *GRIN2A*, or *GRIN2B* regarding their improvements in (A) behavior, (B) development, and (C) seizure frequency. Twelve of 17 (71%) improved in behavior, six of 12 (50%) improved in development, and seven of 18 (39%) had reduced seizure frequency. Seizure frequency reduction of these seven individuals is shown in detail. For Individual #7 the bar is dashed, due to unknown percentage of seizure frequency reduction.

Figure 2 shows the analysis of the total cohort (*n* = 19) with GoF variants. Off‐label memantine treatment was associated with clinical improvements in 14 of 19 individuals (74%). Individuals #3, #7, #11, and #22 appeared to develop tolerance to memantine over time, forming a third subgroup with an initial positive effect. The positive effects seen in the affected individuals plateaued after a short period of time. All of them were recorded as having initial behavioral improvements. No information is available on the effects of the discontinuation of memantine, nor about how soon the behavioral improvements were noticed for this subgroup.

Among the 19 individuals with GoF variants, information on behavioral disorders was available in 17 cases, showing improvements following memantine treatment in 12 of 17 cases (71%). Parents and clinicians reported an increase in awareness and concentration as well as reduced restlessness in eight cases (see Table S1). Mostly improvements in social interactions were mentioned and in particular sleep behavior changes, for example, fewer nighttime awakenings and improved sleeping through the night. In particular, Individual #21^11^ showed fewer arousals during the night. Moreover, two cases stated improved sleeping patterns (Individuals #3 and #26), or no specification regarding the detailed behavioral improvement was made (Individuals #5 and #14). No behavioral improvements were observed in five individuals, and for two cases no data regarding behavior were available (see Table S1 and Figure 2A).

Data on developmental milestones were available for 12 cases, showing improvements in six (50%). Advances were seen in communication skills and cognition, as well as in motor skills. Examples are Individual #21, who markedly improved in his walking abilities, and Individual #5, who developed improved communication skills as well as mobility. Individual #11 had relevant improvements in coordination, particularly of head and eye movements. No developmental improvements were observed in six individuals, and for seven cases, no data were available (see Table S1 and Figure 2B).

The majority of individuals with GoF variants (18/19, 95%) were diagnosed with epilepsy. In seven of these 18 (39%), memantine treatment was associated with reduced seizure burden. Individual #14 became seizure‐free after initially having had monthly seizures. Individual #27 had an almost 95% reduction of epileptic spasms, with a drop from 150–200 down to 7–14 spasms per week and from 10–20 tonic–clonic seizures down to 3–5 seizures per week.^8^ Individual #26 had an 80% reduction of seizure frequency.^12^ Individual #20 had a 70% reduction of seizure frequency, with an average drop from 11 down to three seizures per week.^9^ A 59% seizure frequency reduction could be detected in Individual #33.^10^ Individual #34 experienced a 50% seizure frequency reduction, with one generalized seizure per week dropping down to one seizure every 2 weeks, also associated with a reduction of seizure length from 5–10 min down to 3 min. Individual #7 experienced a reduced seizure frequency of unknown percentage (Figure 2C). In eight of 18 individuals with epilepsy, memantine treatment had no effect on seizure frequency (see Table S1), including two cases (Individuals #3 and #11) who had already become seizure‐free due to conventional antiseizure medication prior to memantine treatment. In three individuals (#10, #25, and #32), seizure frequency worsened during memantine treatment; however, the exact cause and the extent of the seizure exacerbation remained unclear in these three cases. Individual #10 was only treated for 4 days with memantine. It is of note that #10 carried the identical variant as #27, who benefited from memantine treatment. For Individual #32, memantine was discontinued after 2 months of treatment due to worsening of seizure frequency as well as EEG pattern. This discontinuation resulted in even more seizures, leading to a reintroduction of memantine, which was well tolerated the second time, although the patient still had multiple seizures.^10^ Also, Individuals #32, #33, and #34 carried the same variant in *GRIN2D*, leading to completely different outcomes regarding the frequency of seizures (#33 and #34 with seizure frequency reduction approximately at 50% and #32 with worsening in seizure frequency).^10^

EEG data were available in 11 of 19 individuals. Among the 11 cases with available data, six showed mild improvements in EEG patterns following memantine treatment (6/11 individuals, 55%). Most improvements comprised a reduction of spikes and an improvement of the background. As an example, in Individual #1, DEE with spike wave activation in sleep^21^ was slightly reduced from 100% to 85%. In Individual #20, EEG still showed a disorganized background, but asymmetries as well as epileptiform discharges during wakefulness and sleep disappeared.^9^ For Individual #26, spikes and waves were significantly reduced with additional clearing of interictal background.^12^ The EEG of Individual #27 improved, as epileptiform discharges were no longer present, despite a remaining mild slowing of background activity.^8^ Three cases had worsening of EEG findings. In Individual #24,^11^ the EEG showed deterioration due to new multifocal epileptic foci after introduction of memantine, and the EEG in Individual #32 demonstrated higher voltages and contained new bifrontal and multifocal discharges, which led to the initial discontinuation of memantine in this case.^10^ Individual #14's EEG showed multifocal discharges on a regular background.

In contrast to individuals with GoF, those with LoF, indeterminate, and no effect variants did not appear to experience significant benefits from memantine treatment. Mean age was 13 years, with equally distributed genders and an average treatment period of 4 months. Among the eight individuals with LoF variants, two experienced potential benefits, comprising mild improvements in behavior such as in alertness and attention (Individuals #9 and #15). Also, Individual #23, considered to have an indeterminant variant, experienced mild improvements in awareness and reduced restlessness. However, these reported improvements should be considered with caution, as they were based solely on parental observations and were not independently confirmed by the treating physicians. All other symptoms remained unchanged and did not improve during treatment with memantine. Individual #2 experienced mild side effects (reported as “numb behavior” such as fewer contacts with social surroundings) during their 7‐week memantine treatment, leading to memantine being discontinued. In addition to individuals with LoF, indeterminate, or no effect variants, we also identified five cases with VUSs and one with a likely benign variant, which argue against a GRIN‐related disorder. According to the records of these five individuals, nevertheless two had reduced seizure frequency or even became seizure‐free after memantine treatment (see Table S1).

Fisher exact test was used to assess the association between GoF and LoF variants. A significance level of .05 was applied.^22^ We observed improvements in 14 of 19 individuals with GoF variants, whereas only three of 15 individuals with LoF or indeterminate variants improved (14/19 GoF vs. 3/15 LoF, *p* = .0049). Significant differences were observed in behavioral (12/17 GoF vs. 3/15 LoF, *p* = .006), EEG (6/11 GoF vs. 1/13 LoF, *p* = .0233), and developmental improvements (6/12 GoF, 1/14 LoF, *p* = .0261). However, memantine‐induced improvement in reduction of seizure frequency (7/18 GoF vs. 2/10 LoF, *p* = .417) did not reach significance, although still it was more prevalent among individuals with GoF variants.

### 3.5 Localization, distance to binding site, and memantine IC_50_

**FIGURE 3:**
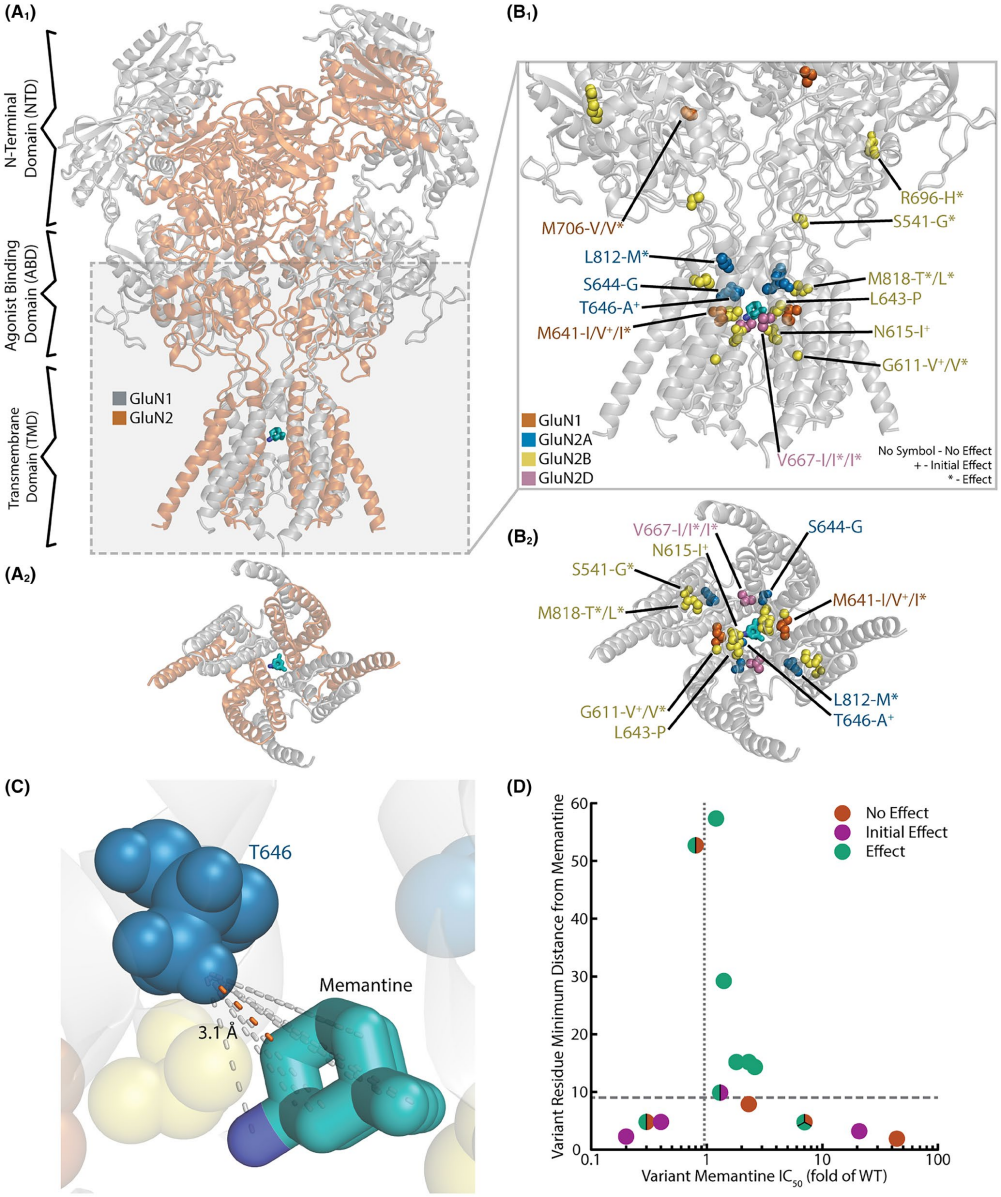
Spatial considerations of the gain‐of‐function (GoF) variants on memantine treatment. (A) A pair of images (full side view, A_1_; isolated transmembrane domain view from the intracellular side of the pore, A_2_) of the N‐methyl‐D‐aspartate receptor structure (GluN1 subunits in gray, GluN2 subunits in red),^6^, ^23^, ^24^ highlighting the binding site of memantine (teal) in the channel pore. (B_1,2_) Location of the GoF de novo variant residues (orange, *GRIN1*; blue, *GRIN2A*; yellow, *GRIN2B*; pink, *GRIN2D*) in relation to memantine. Each individual is represented in the image, where “*” next to the variant residue denotes that an individual had a memantine effect, “+” denotes an individual that had an initial/habituated memantine effect, and for residues without a sign, no memantine effect was measured. (C) Representative image illustrating the method used to measure the minimum residue distance to memantine in its binding site. Similar measurements were made for each atom in a residue, and the overall minimum distance was used. (D) Binary classification of memantine efficacy stratified by memantine IC_50_ (fold of wild‐type [WT] control), using a cutoff of 9 Å confers the optimal classification.

To consider the potential spatial impacts of GRIN variants on memantine binding and memantine efficacy, we analyzed the GRIN genes with the respective variants in a three‐dimensional structural model,^23^, ^24^ in reference to memantine bound in the channel pore (colored turquoise, Figure 3A). Figure 3B illustrates the investigated set of GoF variants in *GRIN1* (orange), *GRIN2A* (blue), *GRIN2B* (yellow), and *GRIN2D* (pink). Most of the GoF variants (14/19 individuals, 10/14 variants) are located in the transmembrane domain (TMD), which is where the memantine binding site is located. Two variants (three individuals: #5, #13, and #14) are located in the agonist‐binding domain (ABD) consisting of the S1 and S2 regions in the polypeptide chain. Two variants are located in the ABD‐TMD linkers (S1‐M1 [#1], and S2‐M4 [#20]), elements critical for receptor gating.^25^ On average, the GoF variants are located 16 ± 18 Å (SD) from bound memantine, which is similar to the mean distance to memantine for the LoF variants (17 ± 4.5 Å, SD). In this limited dataset, none of the LoF variants lies as far from the memantine binding site as the most distal GoF variants (Figure 3C,D). LoF and GoF variants can be found throughout the receptor.^26^

The “effect” and “no effect” groups of variants have similar ranges of distance from memantine ("effect" group, mean minimum displacement = 21 ± 19.0 Å; no effect group, mean minimum displacement = 14 ± 22 Å; mean ± SD). The “initial effect” group is much closer (5.0 ± 3.4 Å) but is too small of a dataset to consider (*n* = 4).

We examined how the distance of each variant from the memantine‐binding pocket correlates with functional parameters (Figure 3D) and uncovered a noteworthy trend. We identified that the memantine IC_50_ (fold of WT control) of variants had a wider spread of values when they were closer to memantine (seven variants <9 Å away from memantine, memantine log fold IC_50_ SD = .86; 11 variants >9 Å away from memantine, memantine log fold IC_50_ SD = .17). Three of the nine variants >9 Å away from memantine (3/7 unique variants) had significantly different memantine IC_50_ values (minimum IC_50_ .84‐fold of WT, maximum IC_50_ 2.6‐fold of WT), whereas all 10 of the variants <9 Å away from memantine (seven unique variants) had significantly different memantine IC_50_ values (minimum IC_50_ .23‐fold of WT, maximum IC_50_ 44‐fold of WT; Fischer exact test, *p* = .0031). Thus, proximity to the memantine binding pocket tends to amplify the effect of a variant—producing markedly higher or lower IC_50_ values—whereas more distant variants generally yield IC_50_ values closer to the WT receptor. This suggests some variants near the memantine binding site alter its interaction with the pore‐lining residues and may significantly alter its potency.

In addition to memantine IC_50_, the clinical efficacy of memantine to treat individuals with GoF variants varied depended on how near the variant was to the memantine binding site. Of the nine variants >9 Å away from the memantine binding site, seven had a positive effect with memantine treatment, one variant had an initial effect with memantine treatment, and one variant had no effect with memantine treatment. Of the 10 variants <9 Å away from the memantine binding site, three variants had a positive effect with memantine treatment, three variants had initial effects with memantine treatment, and four variants had a negative or no effect with memantine treatment. Utilizing the 9‐Å cutoff produces the optimal binary classification to predict the individuals that had clinical efficacy of memantine based on their variant residue's distance from the memantine binding site. In this classification, each individual is scored (i.e., redundant variants are counted separately) and only those individuals who had therapeutic effects by memantine administration are considered as a positive result (initial effect and no effect are classified as negative, seven true positives, seven true negatives, three false negative, and two false positives; Fisher exact test, *p* = .0698). If we consider individuals with the same variant and instead ask whether there has been at least one observed positive memantine effect for any of particular variant occurrences (i.e., is a therapeutic benefit possible for a given residue), the classification reaches the significance threshold (e.g., if one individual had a memantine effect and the other did not, we consider this variant as a positive effect; seven true positives, five true negatives, two false negatives, and zero false positives; Fisher exact test, *p* = .0210).

## 4 DISCUSSION

We demonstrate that, in several published and unpublished individuals, the rationale for the clinical use of memantine was not appropriately informed by the pathogenic and functional classification of the detected GRIN variants. Consequently, the previous known and published spectrum of memantine treatment responses appears diverse and inconsistent. Our study reveals a distinct and significant positive treatment effect of memantine in individuals with truly pathogenic GoF GRIN variants.

A therapeutic benefit of the NMDAR channel blocker memantine was seen in 74% of individuals with GoF GRIN variants and was most notable in behavioral improvements (71%), followed by developmental progress (50%) and reduction of seizure frequency (39%).

The broad spectrum and pattern of improvements mirrors the observations in individuals with LoF GRIN variants who underwent an NMDAR coagonistic treatment via L‐serine, who also showed particularly behavioral improvements (89% of patients), followed by developmental progress (44% of patients) and reduction of seizure frequency and/or improvement of EEG (44% of patients).^4^, ^5^, ^27^ Four individuals with GoF GRIN variants who received memantine treatment experienced a memantine‐related worsening of seizure frequency (Individuals #10, #25,^16^ and #32^10^) and/or EEG (Individuals #24^11^ and #32^10^). In all other individuals with GoF GRIN variants, memantine appeared to be tolerated well. Individuals #3 and #21,^11^ carrying the same variant c.1832G>T, p.(Gly611Val) in *GRIN2B*, were treated at approximately the same age (~8 years old), resulting in similar improvements in behavior and development. However, in Case #3, the effects of memantine plateaued after several months, which was not seen in Individual #21. For Cases #10 and #27^8^ (c.1923G>A. p.[Met641Ile] in *GRIN1*), as well as #32, #33,^10^ and #34 (c.1999G>A, p.[Val667Ile] in *GRIN2D*), results regarding memantine treatment were the opposite. In Cases #32 and #33, differences in the age at treatment onset may have contributed to the varying outcomes of memantine therapy. Individual #32 started treatment at 6 years 6 months of age, whereas individual #33 started at 2 years 6 months. In contrast, the age difference for Cases #10 and #27 was only 7 months (Individual #10 started at 7 months and #27 at 14 months). However, the treatment duration varied greatly; Individual #10 received memantine for only 4 days, which is not comparable to the 14‐month treatment duration in Individual #27. Additionally, Individuals #13 and #14, carrying the variant c.2116A>G, p.(Met706Val) in *GRIN1*, had inhomogeneous results. Case #13 did not improve through treatment with memantine for an unknown duration, whereas Individual #14 responded well, with improvements in behavior and development within a treatment period of 9 months. Conclusively, the sample size is too small to draw meaningful comparisons between individuals carrying the same variant, regarding treatment response and benefits from memantine treatment.

Memantine treatment in individuals with LoF GRIN or indeterminate variants did not usually result in exacerbation of the phenotype, as only one individual (#2) experienced mild worsening of their behavioral disorder. This was unexpected, as we previously observed an exacerbation of the behavioral disorder in an individual with a GoF GRIN variant erroneously treated with L‐serine.^28^ Nonetheless, compared to individuals with GoF GRIN variants, the benefits of memantine treatment were significantly less frequent among those with LoF GRIN, indeterminate, or no effect variants. Parents reported mild improvements of cognition, behavior, and alertness during memantine treatment in only three individuals (#9, #15, and #23) with LoF or indeterminate variants. However, these observations were not corroborated by the treating physicians. Given the nature of the variants, it is likely that these improvements were unrelated to the underlying genetic cause.

Thus, an objective beneficial treatment response to memantine in individuals with GRIN‐related disorders can solely be expected in the case of GoF variants. However, not all GoF variants appear to be similarly responsive. Therefore, we investigated whether the distance from the pathogenic GRIN variant to the memantine binding site as well as the sensitivity to memantine might influence memantine binding affinity and thus, therapeutic efficacy. We observed that GoF variants further away from the memantine binding site are more likely to retain a WT‐like memantine potency and may be more clinically efficacious. Additionally, variants that are close to the memantine binding site (<9 Å) typically have altered memantine potency (either enhanced or diminished) and are less likely to have clinical effectiveness. GoF variants that do not deviate from WT memantine sensitivity may provide an opportunity to mitigate actions of variant receptors via preferential block by memantine. We could not identify a significantly higher prevalence of variants that are more sensitive to memantine in Group 1 compared to Group 3, but we observed a slightly higher prevalence of GoF variants that lose sensitivity (right shift in 3/5 variants) in Group 3. Presumably, the groups are too small to identify robust differences.

In conclusion, our study underlies the importance of a correct classification of the respective variant prior to making treatment decisions. Additionally, our data highlight the distance to memantine binding site as a potential predictor for a positive treatment outcome with memantine in individuals with GoF GRIN variants. Standardized developmental assessments were not conducted in the individuals studied, either before or during memantine treatment. The information is based on observations by the treating physicians and parents. Furthermore, this was not a prospective study with a blinded crossover design and defined washout periods. In light of these limitations, further clinical trials are of utmost importance to reliably assess the efficacy and safety of memantine in this context.

## AUTHOR CONTRIBUTIONS

The authors A.S. Schoonjans, D.C.M Veenma, I. Borggraefe, K. Park, M. Somorai, P. Striano, S. Syrbe, T.A. Fantaneanu and T. Benke participated in the clinical care of the investigated individuals and contributed relevant clinical information. Oocyte recordings were performed by S.J. Myers and E. McDaniels, and HEK cell recordings were performed by R.E. Perszyk. S.J. Myers, R.E. Perszyk, W. Chen, S.F. Traynelis, and H. Yuan were involved in experimental design. Data was analyzed by M. Karnstedt, I. Krey, R.E. Perszyk, and H. Yuan. M. Karnstedt, I. Krey, J.R. Lemke, R.E. Perszyk, S.F. Traynelis, and H. Yuan wrote the manuscript. All authors, mainly I. Krey, were involved in revising the manuscript.

## CONFLICT OF INTEREST STATEMENT

J.R.L., T.A.B., and S.F.T. report financial support was provided by Simons Foundation. J.R.L., T.A.B., and S. F.T. report a relationship with the Simons Foundation that includes funding grants. Support was provided by NIH‐NINDS (NS111619, S.F.T.), NIH‐NICHD (HD082373, H.Y.), the GRIN2B Foundation (H.Y.), Austin's Purpose (S.F.T.), and Imagine, Innovate, and Impact Awards from the Emory University School of Medicine and through the Georgia CTSA NIH award (UL1‐TR002378, H.Y.). The content of this publication does not necessarily reflect the views or policies of the Department of Health and Human Services, nor does the mention of trade names, commercial products, or organizations imply endorsement by the US Government. The authors declare the following competing interests: S.F.T. is a member of the scientific advisory board for EuMentis Therapeutics and Neurocrine Biosciences, a consultant for Seyltx, a cofounder of NeurOp and AgriThera, and a member of the board of directors for NeurOp. S.F.T. is principal investigator (PI) of a study receiving a grant from GRIN Therapeutics. H.Y. is PI of a study receiving a grant from Sage Therapeutics. S.F.T., J.R.L., T.A.B., and K.P. are members of the medical advisory board for the GRIN2B Foundation and the CureGRIN Foundation and consultants for GRIN Therapeutics. The other authors declare that they have no known competing financial interests or personal relationships that could have appeared to influence the work reported in this paper.

## ETHICS STATEMENT

We confirm that we have read the journal's position on issues involved in ethical publication and affirm that this report is consistent with those guidelines. Furthermore, this research was approved by the ethics committee of the University of Leipzig under the signature 379/21‐ek. All in vitro studies were conducted according to the guidelines of Emory University School of Medicine.

## DECLARATION OF GENERATIVE AI AND AI‐ASSISTED TECHNOLOGIES IN THE WRITING PROCESS

During the preparation of this work, the authors used ChatGPT to improve language and readability. After using this tool, the authors reviewed and edited the content as needed and take full responsibility for the content of the publication.

## Supporting information


Table S1.

Data S1.

## Data Availability

The data that support the findings of this study are available from the corresponding author upon reasonable request.
