# Supplementary material for: Memantine treatment in individuals with GRIN gain‐of‐function variants is associated with improvements in behavior, development, and seizure frequency
Source: Epilepsia. 2026 Jan 5;67(4):1961–74. doi: 10.1002/epi.70090 (PMC13075606; doi:10.1002/epi.70090)
Supplement: Supplementary file 2 — Data S1. [file EPI-67-1961-s001.docx]

**Supplementary Material and Methods**

*Xenopus* oocytes preparations and injections

The plasmid (pcIneo) harboring the cDNA was purified and linearized, and RNA was synthesized *in vitro* (Ambion mMESSAGE mMACHINE). Stage V-VI *Xenopus laevis* oocytes were obtained from commercial vendors as previously described ^(1–11)^. Unfertilized *Xenopus laevis* oocytes within ovaries were obtained from a commercial vendor (Xenopus One, Inc), digested with Collagenase Type 4 (Worthington-Biochem, Lakewood, NJ, USA) at a concentration of 800 μg/ml in Ca^2+^-free Barth's solution made of (in mM) 88 NaCl, 2.4 NaHCO_3_, 1 KCl, 0.82 MgSO_4_, and 10 HEPES (pH 7.4 with NaOH), supplemented with 100 μg/ml gentamycin, 1 U/ml penicillin, and 1 μg/ml streptomycin. The ovary was placed in enzyme with gentle mixing (23°C) for two hours. Oocytes were rinsed 10 times with Ca^2+^-free Barth's solution (~40 ml each time) for five minutes, and rinsed four more times with normal Barth's solution that included 0.41 mM CaCl2 and 0.33 mM Ca(NO_3_)_2_ on a slow shaker. Wild type (WT) and variant GluN1:GluN2A RNA (ratio 1:2) was injected into *Xenopus laevis* oocytes and the oocytes were incubated at 16°C for 2–3 days. A total of 0.25‐10 ng of RNA in 50 µl of RNAase-free water was injected per oocyte. Oocytes were maintained in normal Barth’s solution at 16°C.

Two electrode voltage (TEVC) clamp recordings from oocytes

Each variant was functionally analyzed in oocytes to measure the glutamate EC_50_ and glycine EC_50_ at -40 mV, Mg²⁺ IC_50_ at -60 mV, and MTSEA open channel probability at -40 mV. Additionally, the IC_50_ value for memantine block was determined at -40 mV, in the presence of maximal glutamate and glycine (100 μM each) receptor activation, to assess if the missense variant altered memantine inhibition ^(12)^.

Two-electrode voltage clamp recordings were performed as previously described ^(1)^. A dual‐stage micropipette puller was used to prepare the microelectrodes from borosilicate glass with resistance of 4-8 MOhm (TW150F‐4; World Precision Instruments, Sarasota, FL). Current and voltage electrodes were filled with 0.3 or 3 M KCl, respectively. Oocytes were placed in a multi-track recording chamber that shared a single perfusion line, allowing simultaneous recordings. Oocytes expressing recombinant NMDARs were perfused with solution containing (in mM) 90 NaCl, 1.0 KCl, 0.5 BaCl_2_, 10 HEPES, and 0.01 EDTA adjusted to pH 7.4 with NaOH (23˚C). For Mg^2+^ potency studies, EDTA was omitted from the recording solution.

To assess the glutamate potency, the oocytes were held under voltage clamp at -40 mV unless indicated otherwise, and oocytes were superfused with buffer including sequentially increasing concentrations of L-glutamate (6-7 concentrations) for 0.75-minute duration each in the continuous presence of 100 µM glycine to obtain concentration-response data. Glutamate concentrations were selected to achieve maximal activation at the highest concentration. If variants studied reduced the glycine potency, the concentration of glycine was increased to at least 10 times higher than the variant glycine EC_50_. Results at each glutamate concentration are normalized to the maximum receptor activation levels achieved (defined as 100%). The data are fitted by equation 1 to obtain an EC_50_ value for each oocyte.

*Response* (%) = 100/(1+ (EC_50_ / [*agonist*])^nH^), Equation 1

EC_50_ = agonist concentration that elicited a half maximal response
nH = Hill slope

To assess glycine potency, oocytes were similarly superfused with buffer including increasing concentrations of glycine (6-7 concentrations) in the presence of a maximally effective concentration of glutamate to measure the concentration-response relationship. Glycine concentrations were selected to achieve maximal activation at the highest concentration. The data were fitted by Equation 1 to obtain an EC_50_ value for each oocyte.

To assess Mg^2+^ potency, oocytes were held at -60 mV. After a stable baseline was achieved, oocytes were activated by maximally effective concentrations of L-glutamate and glycine, followed by increasing concentrations of Mg^2+^ co-applied in the continuous presence of maximal glutamate and glycine. The responses at each Mg^2+^ concentration were normalized to the maximum receptor activation without Mg^2+^ (set to 100%) and the IC_50_ values obtained by fitting concentration-inhibition data with

*Response* (%) = (100 - *minimum*)/(1+ ([Mg^2+^] / IC_50_)^nH^) + *minimum* Equation 2

*minimum* = residual percent response in saturating concentration (constrained to be > 0) of Mg^2+^IC_50_ = concentration of Mg^2+^ that causes half maximal inhibition
nH = Hill slope
Inhibition at 1 mM Mg^2+^ was taken from the data or calculated from fitted IC_50_ and nH

To assess open probability, variant GluN1 subunits were co-expressed with GluN2A-A650C or GluN2B-A651C and variant GluN2 subunits were expressed with GluN1-A652C cRNA at a GluN1:GluN2 ratio of 1:2. The oocyte was held at -40 mV and oocytes were superfused with buffer including a maximally effective concentration of glutamate and glycine for one minute, after which the solution was switched for three minutes to one in which maximally effective concentrations of agonists were supplemented with 0.2 mM of the covalent modifying reagent 2-aminoethyl methanethiolsulfonate hydrobromide (MTSEA; Toronto Research Chemicals, Ontario, Canada), which was prepared fresh and used within 30 minutes. The channel open probability (P_OPEN_) was estimated from the fold potentiation observed in MTSEA according to

Open probability = (γ_MTSEA_ / γ_CONTROL_) × (1 / Potentiation) Equation 3

γ_MTSEA_ and γ_CONTROL_ = single channel chord conductance values estimated from GluN1/GluN2A receptors
Potentiation = ratio of current in the presence of MTSEA to current in the absence of MTSEA; γ_MTSEA_ / γ_Agonist_ was 0.67 ^(13)^.

Variants residing within one helical turn of the sites MTSEA modifies (GluN1-A652, GluN2A-A650, GuN2B-A651) might alter this assay and results for these few variants should be interpreted cautiously.

Whole cell patch clamp recordings from HEK293 cells

Human embryonic kidney (HEK) 293 cells (ATCC CRL-1573) plated on 12 mm glass coverslips precoated with 0.1 mg/ml poly-D-lysine were grown in Dulbecco’s Modified Eagle Medium (Gibco 10569-010, DMEM + GlutaMAX) supplemented with 10% dialyzed fetal bovine serum and 10 U/ml penicillin and 10 µg/ml streptomycin at 37°C in humidified 5% CO_2_. Cells were transiently transfected with calcium phosphate and cDNA at 0.5 μg/well total cDNA encoding GluN1:GluN2:eGFP ratios and incubated with 200 μM DL-APV and 200 μM 7-CKA for 12-24 hours prior to whole cell patch clamp recording of glutamate-activated NMDAR currents, as previously described ^(1)^. Briefly, the chamber was perfused with recording solution comprised of (in mM) 3 KCl, 150 NaCl, 0.01 EDTA, 1.0 CaCl_2_, 10 HEPES, and 22 D-mannitol (pH 7.4 with NaOH). The patch electrodes (resistance 3-5 MΩ) were made from thin-walled glass micropipettes (TW150F-4, World Precision Instruments, Sarasota, FL, USA) and filled with internal solution comprised of (in mM) 110 D-gluconate, 110 CsOH, 30 CsCl, 5 HEPES, 4 NaCl, 0.5 CaCl_2_, 2 MgCl_2_, 5 BAPTA, 2 NaATP and 0.3 NaGTP (pH 7.4 with CsOH; 300-305 mOsmol/kg). Whole cell current responses to maximally-effective concentrations of glutamate (1 mM) and glycine (100 µM) at -60 mV were recorded (23°C). Current responses were low pass filtered (8 kHz, 8-pole Bessel -3 dB; Frequency Devices) and digitized at 20 kHz (Digidata 1440A; Molecular Devices) by Clampex 10.3 (Molecular Devices). The position of a two-barreled theta-glass micropipette used for rapid solution exchange was controlled by a piezoelectric translator (Siskiyou, Grants Pass, OR, USA) such that the cell was exposed to agonist for either 2-8 milliseconds or for 1 second. Data were filtered off-line (2 kHz -3 dB) and the deactivation time course fitted by a dual exponential function

Response=Amplitude_FAST_(exp(-time/tau_FAST_))+Amplitude_SLOW_(exp(-time/tau_SLOW_)) Equation 4

The weighted deactivation tau τ_W_ was calculated by

τ_W_ = (Amplitude_FAST_ tau_FAST_ +Amplitude_SLOW_ tau_SLOW_ ) / (Amplitude_FAST_ + Amplitude_SLOW_) Equation 5

Current responses for some cells with larger amplitudes were corrected for series resistance filtering off-line ^(14)^ prior to fitting.

Surface protein assay

We assayed beta-lactamase (β-lac) activity in cells transfected with NMDAR variant cDNAs encoding the β-lac open reading frame fused in-frame at the end of the signal peptide sequence for GluN1, GluN2A, or GluN2B subunits so that active β-lac enzyme faced the extracellular solution ^(4, 5, 8)^. HEK cells were plated in 96-well plates at 50,000 cells/well in serum-supplemented DMEM and transiently transfected 24 hours later with cDNA encoding β-lac-GluN1 variants with WT GluN2, or β-lac-GluN2 variants with WT GluN1 using Fugene6 (Promega, Madison, WI). Wells transfected with Fugene6 alone (no cDNA) defined background absorbance. Immediately after transfection NMDAR antagonists (200 μM DL-APV and 200 μM 7-CKA) were added to each well. 24 hours later cells were rinsed with Hank’s Balanced Salt Solution (HBSS) containing (in mM) 140 NaCl, 5 KCl, 0.3 Na_2_HPO_4_, 0.4 KH2PO_4_, 6 glucose, 4 NaHCO_3_ and supplemented with 10 mM HEPES (pH 7.4). Subsequently, 100 μl of 100 μM nitrocefin (Millipore, Burlington, MA, USA) in HBSS with HEPES was added to un-lysed wells used for measuring the surface level of NMDAR surface expression. To lyse wells, we added 50 μl H_2_O for 30 minutes, then triturated the cells briefly, and then added 50 μl of 200 μM nitrocefin to determine the total enzymatic activity, which reflects the total NMDAR subunit protein expression level. The absorbance at 486 nm was measured each minute for 30 minutes at 30°C using a microplate reader. The rate of increase in absorbance was generated from the slope of a linear fit to the data for each well as previously described ^(1)^.

Residue spatial analysis

To determine the distance of individual variant residues from memantine in its binding site in the NMDAR pore, the sequences for GluN1, GluN2A, GluN2B, GluN2C, GluN2D were aligned using multi-sequence alignment using phylogenic tree distances (Matlab functions, seqneighjoin and multialign). Analogous GluN2B residues were determined for variants in GluN2A. Three-dimensional coordinates for residues were taken from a GluN1/GluN2B-model ^(15)^ based on the non-active GluN1/GluN2B structure (6WHS, ^(16)^). Memantine coordinates were taken from the memantine-bound GluN1/GluN2B structure (7sad, ^(17)^), after alignment with the previous model, since this structure residue side chains are not fully modelled. For each variant, the full set of atomic vector norm distances between all wild-type residue atoms (main chain and side chain) and all memantine atoms were determined and the lowest distance was taken.

**Supplementary Table 1**: Summary of patients and variants information (*see* attached Excel file)

**Supplementary Table 2** Classification of functional variant data by the criteria of *Myers et al.,* (2023) ^(1)^

| ***Individual*** | ***Gene*** | **Variant** | **Glutam. EC_50_** | **Glycine EC_50_** | **Mg^2+^ IC_50_** | **P_OPEN_** | **τ_WEIGHT_** | **Surface** | **Count High, Mod** | **Charge Transfer**  Synaptic, Non-Synaptic | **Class** | **PMID Data** |
| --- | --- | --- | --- | --- | --- | --- | --- | --- | --- | --- | --- | --- |
| ***#30*** | ***GRIN1*** | **p.(Ser560dup)** | **tstm** | **tstm** | **tstm** | **H (0.017)** | **tstm** | 0.99 | **5, 0** | **tstm, tstm** | **Likely LoF** | 37000222 |
| ***#9*** | ***GRIN1*** | **p.(Gly618Ser)** | **M (0.56)** | **M (0.46)** | 1.2 | **H (0.18)** | 1.0 | 1.1 | **1, 2** | **0.39, 0.15** | **Likely LoF** | This study |
| ***#10, #27*** | ***GRIN1*** | **p.(Met641Ile)** | 1.1 | **M (1.7)** | **H (8.1)** | **H (0.35)** | 1.2 | 0.71 | conflict | **3.6, 2.2** | **Possible GoF** | 34227748 |
| ***#11*** | ***GRIN1*** | **p.(Met641Val)** | 1.3 | 1.4 | **M (1.5)** | **M (0.57)** | 0.96 | 1.3 | conflict | **1.8, 2.6** | **Possible GoF** | This study |
| ***#12*** | ***GRIN1*** | **p.(Ala652Thr)** | 0.87 | 1.2 | 1 | **H (0.50)** | 0.71 | 1 | **1, 0** | **0.35**, **0.52** | **Likely LoF** | 38538865 |
| ***#13, #14*** | ***GRIN1*** | **p.(Met706Val)** | **M (1.6)** | 0.69 | **M (1.6)** | 0.84 | 0.96 | 1.2 | **0, 2** | **1.1, 2.7** | **Possible GoF** | This study |
| ***#16*** | ***GRIN1*** | **p.(Gly815Arg)** | **H (0.26)** | **M (0.5)** | H (5.5) | **H (0.061)** | **H (0.48)** | 0.86 | conflict | **0.086, 0.038** | **Possible LoF** | This study |
| ***#15*** | ***GRIN1*** | **p.(Gly815Trp)** | **M (1.7)** | **M (1.5)** | **H (3.1)** | **H (0.024)** | **M (1.8)** | 0.86 | conflict | **0.17, 0.16** | **Possible LoF** | This study |
| ***#17*** | ***GRIN1*** | **p.(Gly827Arg)** | **tstm** | **tstm** | **tstm** | **tstm** | **tstm** | **H (0.066)** | 6, 0 | **tstm, tstm** | **Likely LoF** | This study |
| ***#18*** | ***GRIN1*** | **p.(Arg844Pro)** | 1.4 | 1.3 | 1 | 1.4 | 1.3 | 1.1 | subthreshold | **2.3, 4.2** | **No Effect** | This study |
| ***#31*** | ***GRIN1*** | **p.(Lys1045_Ser1050 delinsAsn)** | ND | ND | ND | ND | ND | ND | ND | **ND** | **ND** | - |
| ***#28*** | ***GRIN2A*** | **p.(Leu361=)** | ND | ND | ND | ND | ND | ND | ND | **ND** | **ND** | - |
| ***#6*** | ***GRIN2A*** | **p.(Ser644Gly)** | **H (20)** | **H (17)** | **M (1.7)** | **H (5.2)** | **H (39)** | **H (0.34)** | conflict | **>100, >100** | **Possible GoF** | 38538865 |
| ***#7*** | ***GRIN2A*** | **p.(Thr646Ala)** | **H (25)** | **H (16)** | **M (1.8)** | **H (3.4)** | **H (47)** | **H (0.041)** | conflict | **8.2, 6.7** | **Possible GoF** | 38538865 |
| ***#20*** | ***GRIN2A*** | **p.(Leu812Met)** | **H (8.7)** | **H (14)** | **M (2.0)** | **H (2.2)** | **H (9.4)** | 0.85 | 4, 1 | **32, 49** | **Likely GoF** | 39535073 |
| ***#29*** | ***GRIN2A*** | **p.(Leu963Pro)** | ND | ND | ND | ND | ND | ND | ND | **ND** | **ND** | - |
| ***#8*** | ***GRIN2A*** | **p.(Pro1199Argfs*32)** | ND | ND | ND | ND | ND | ND | ND | **ND** | **ND** | - |
| ***#1*** | ***GRIN2B*** | **p.(Ser541Gly)** | **H (4.3)** | **H (4.8)** | 1.1 | **M (1.8)** | **H (2.3)** | M (0.60) | conflict | **1.8, 4.4** | **Possible GoF** | 37369021 |
| ***#2*** | ***GRIN2B*** | **p.(Ser555Ile)** | **tstm** | **tstm** | **tstm** | **tstm** | **tstm** | 1.0 | **5, 0** | **tstm, tstm** | **Likely LoF** | 37369021 |
| ***#3, #21*** | ***GRIN2B*** | **p.(Gly611Val)** | 0.88 | **M (1.5)** | **H (>100)** | **M (0.54)** | **M (1.5)** | 1.2 | conflict | **28, 12** | **Possible GoF** | 37369021 |
| ***#22*** | ***GRIN2B*** | **p.(Asn615Ile)** | 1.2 | **M (2.0)** | **H (>100)** | **M (0.54)** | 0.88 | 0.79 | conflict | **17, 27** | **Possible GoF** | 37369021 |
| ***#23*** | ***GRIN2B*** | **p.(Val618Gly)** | 0.79 | 0.80 | **H (13)** | **H (0.22)** | 1.4 | 0.72 | conflict | **1.8, 1.0** | **Indeterminant** | 31429998 |
| ***#25*** | ***GRIN2B*** | **p.(Leu643Pro)** | **H (3.0)** | **H (2.9)** | **M (2.2)** | **M (5.1)** | **M (1.9)** | **H (0.12)** | conflict | **1.6, 5.4** | **Possible GoF** | This study |
| ***#4*** | ***GRIN2B*** | **p.(Glu657Asp)** | **H (4.0)** | **M (2.1)** | 1.0 | **M (0.40)** | **H (3.5)** | 1.0 | conflict | **1.7, 2.4** | **Indeterminant** | 37369021 |
| ***#5*** | ***GRIN2B*** | **p.(Arg696His)** | **H (4.5)** | 0.9 | 0.93 | 1.0 | **H (3.6)** | 0.75 | 2, 0 | **1.5, 4.8** | **Likely GoF** | 37369021 |
| ***#26*** | ***GRIN2B*** | **p.(Met818Leu)** | **M (1.8)** | **M (1.8)** | **M (1.9)** | **H (7.4)** | 1.4 | **H (0.49)** | conflict | **7.6, 13** | **Possible GoF** | This study |
| ***#24*** | ***GRIN2B*** | **p.(Met818Thr)** | **H (2.5)** | **M (2.2)** | 0.68 | **H (3.4)** | **H (2.4)** | **M (0.63)** | conflict | **3.5, 4.3** | **Possible GoF** | 39535073 |
| ***#19*** | ***GRIN2D*** | **p.(Ser1271Leu)** | **M (1.6)** | **H (0.5)** | 1.4 | **M (0.48)** | 0.91 | 0.69 | conflict | **0.38, 0.58** | **Possible LoF** | 31504254 |
| ***#32, #33, #34*** | ***GRIN2D*** | **p.(Val667Ile)** | **M (1.5)** | **M (1.7)** | **M (1.6)** | **H (10)** | **M (1.6)** | 0.83 | **1,4** | **9.1, 8.1** | **Likely GoF** | 27616483 |

Data are mean fold change in parameter, with values greater than 1 indicating changes that produce more current and numbers less than one producing less current. All Fold effects are shown to 2 significant figures.

Data were generated as described in Methods for "This study" or reproduced from the indicated papers and included here to facilitate comparison. The data summary for the new variants (“This study”) was presented in Supplementary Table 4.

“tstm” indicates too small to measure.

**Supplementary Table 3A** Functional variant classification approach: rules from *Myers et al.,* 2023 ^(1)^ and also *Xu et al*., 2024 ^(18)^

| **Likely**  **LoF or GoF** | **Possible**  **LoF or GoF** | **No Detectable Effect** | **Indeterminant** | **Indeterminant (*)** | **Likely LoF (*)** |
| --- | --- | --- | --- | --- | --- |
| · One or more changes with high confidence and no conflicts in direction of change | · Two or more changes with moderate confidence and no conflicts in direction of change | · No detectable functional changes for any parameters | · Conflicting changes in opposite functional direction and a change in synaptic and non-synaptic charge transfer between 0.4 and 2.5-fold | · Where data was collected in all six assays but the current amplitude in HEK cells under voltage clamp deemed too small to determine the tau | · Large decrease in response amplitude that precludes other parameter assessments plus evidence for protein synthesis |
|  | · One change of moderate confidence and >2.5-fold or <0.4 -fold change in synaptic or non-synaptic charge transfer | · Only one change with moderate confidence and a change in synaptic and non-synaptic charge transfer between 0.4 and 2.5-fold |  |  |  |
|  | · Conflicting changes of moderate/high confidence and >2.5-fold or <0.4-fold change in synaptic or non-synaptic charge transfer |  | · Variants deemed in conflict and synaptic and non-synaptic charge transfers were both suprathreshold but in conflict |  |  |

We used the synaptic and non-synaptic charge transfer to re-classify conflicting and subthreshold variants as Possible GoF or Possible LoF. We propose that a change >2.5-fold or <0.40-fold in synaptic or non-synaptic charge transfer should elevate conflicting or subthreshold variants to Possible GoF or Possible LoF ^(1)^.

**Supplementary Table 3B** Thresholds for determination of GoF and LoF from *Myers et al.,* (2023) ^(1)^

| **Variant/WT** | **Support for LoF** | **Support for LoF** | **Support for GoF** | **Support for GoF** |
| --- | --- | --- | --- | --- |
|  | **(High Confidence)** | **(Moderate Confidence)** | **(High Confidence)** | **(Moderate Confidence)** |
| **Glutamate potency ratio *^a^*** | **↓** to 0.40 or more | **↓** to 0.67–0.40 | **↑** to 2.5-fold or more | **↑** to 1.5–2.5-fold |
| **Glycine potency ratio *^a^*** | **↓** to 0.40 or more | **↓** to 0.67–0.40 | **↑** to 2.5-fold or more | **↑** to 1.5–2.5-fold |
| **Mg^2+^ IC_50_ ratio *^b^*** | **↓** to 0.40 or more | **↓** to 0.67–0.40 | **↑** to 2.5-fold or more | **↑** to 1.5–2.5-fold |
| **t_W_ deactivation ratio** | **↓** to 0.50 or more | **↓** to 0.67–0.50 | **↑** to 2-fold or more | **↑** to 1.5–2-fold |
| **Open probability ratio** | **↓** to 0.50 or more | **↓** to 0.67–0.50 | **↑** to 2-fold or more | **↑** to 1.5–2-fold |
| **Surface expression ratio** | **↓** to 0.50 or more | **↓** to 0.67–0.50 | **↑** to 2-fold or more | **↑** to 1.5–2-fold |

^a^ The fold change in variant to WT potency ratio was defined as WT EC_50_/variant EC_50_ because of the reciprocal relationship between potency and EC_50_. That is, an increase in variant EC_50_ value reflects a decrease in agonist potency (promoting LoF), and a decrease in variant EC_50_ value reflects an increase in agonist potency (promoting GoF).

^b^ A decrease in Mg^2+^ IC_50_ value reflects an increase in potency (more Mg^2+^ inhibition, promoting LoF), and an increase in Mg^2+^ IC_50_ value reflects a decrease in potency (less Mg^2+^ inhibition, promoting GoF).

**Supplementary Table 4** Data describing the effects on NMDAR functional properties for variants in Supplementary Table 2

| **GRIN-variant** | **Glutamate EC_50_ (95% CI), mM** | **Glycine EC_50_ (95% CI), mM** | **Mg^2+^ IC_50_ (95% CI), mM** | **P_OPEN_** | **τ_WEIGHT_ (ms)** | **Surface expression** |
| --- | --- | --- | --- | --- | --- | --- |
| **WT N1/2A** | 3.6 (3.1, 4.1) (12) | 1.0 (0.91, 1.2) (11) | 21 (18, 25) (12) | 0.23 ± 0.012 (23) | 39 ± 7.0 (8) | 1.0 (4) |
| **1-G618S/2A** | 6.4 (5.9, 7.0) (12) | 2.2 (2.0, 2.5) (12) | 25 (18, 36) (10) | 0.042 ± 0.0036 (15) | 40 ± 13 (4) | 1.1 ± 0.03 (4) |
| **WT N1/2A** | 3.2 (2.9, 3.6) (12) | 1.2 (1.0, 1.4) (12) | 20 (17, 23) (10) | 0.25 ± 0.011 (16) | 50 ± 3.8 (19) | 1.0 (4) |
| **1-M641V/2A** | 2.5 (2.2, 2.9) (12) | 0.85 (0.73, 0.99) (12) | 31 (27, 35) (12) | 0.14 ± 0.008 (13) | 49 ± 4.3 (5) | 1.3 ± 0.11 (4) |
| **WT N1/2A** | 4.0 (3.7, 4.4) (12) | 1.3 (1.2, 1.5) (10) | 29 (19, 44) (13) | 0.20 ± 0.009 (13) | 61 ± 6.7 (13) | 1.0 (4) |
| **1-M706V/2A** | 2.6 (2.1, 3.2) (13) | 1.9 (1.6, 2.3) (11) | 45 (30, 69) (13) | 0.17 ± 0.008 (14) | 59 ± 2.4 (4) | 1.2 ± 0.14 (4) |
| **WT N1/2A** | 3.3 (2.7, 4.0) (20) | 1.3 (0.94, 1.7) (16) | 18 (15, 21) (15) | 0.26 ± 0.011 (16) | 81 ± 6.9 (11) | 1.0 (4) |
| **1-G815R/2A** | 12 (11, 14) (14) | 2.5 (2.2, 2.9) (10) | 100 (73, 137) (17) | 0.016 ± 0.001 (14) | 38 ± 2.4 (5) | 0.86 ± 0.048 (4) |
| **WT N1/2A** | 3.2 (2.9, 3.6) (12) | 1.1 (0.91, 1.3) (11) | 17 (13, 23) (12) | 0.25 ± 0.013 (18) | 54 ± 4.4 (14) | 1.0 (4) |
| **1-G815W/2A** | 1.9 (1.6, 2.3) (12) | 0.73 (0.65, 0.82) (12) | 53 (36, 77) (8) | 0.006 ± 0.0004 (14) | 96 ± 28 (5) | 0.86 ± 0.042 (4) |
| **WT N1/2A** | -- | -- | -- | -- | -- | 1.0 (6) |
| **1-G827R/2A** | tstm | tstm | tstm | tstm | tstm | 0.066 ± 0.02 (6) |
| **WT N1/2A** | 3.4 (3.0, 3.9) (12) | 1.3 (1.2, 1.5) (14) | 26 (20, 34) (12) | 0.25 ± 0.011 (16) | 61 ± 12 (6) | 1.0 (4) |
| **1-R844P/2A** | 2.4 (2.1, 2.9) (12) | 0.99 (0.89, 1.1) (13) | 27 (22, 34) (13) | 0.35 ± 0.02 (14) | 81 ± 17 (4) | 1.1 ± 0.10 (4) |
| **WT N1/2B** | 1.2 (1.0, 1.3) (10) | 0.31 (0.28, 0.33) (10) | 21 (19, 24) (10) | 0.025 ± 0.002 (24) | 613 ± 38 (17) | 1.0 (8) |
| **2B-L643P** | 0.38 (0.33, 0.44) (10) | 0.10 (0.088, 0.12) (10) | 47 (40, 54) (8) | 0.13 ± 0.007 (16) | 972 ± 162 (5) | 0.12 ± 0.033 (6) |
| **WT N1/2B** | 0.91 (0.84, 0.99) (12) | 0.37 (0.32, 0.43) (11) | 38 (27, 53) (10) | 0.019 ± 0.0011 (10) | 505 ± 37 (9) | 1.0 (4) |
| **2B-M818L** | 0.50 (0.42, 0.58) (12) | 0.21 (0.17, 0.25) (12) | 74 (49, 113) (10) | 0.14 ± 0.006 (14) | 722 ± 144 (4) | 0.49 ± 0.023 (4) |

* WT values were recorded on the same day for every variant, and the mean for each parameter is given.

All data are from 8-24 oocyte, or 4-17 HEK cell experiments.

“tstm” indicates the response was too small to measure.

**Bibliography**

1. Myers SJ, Yuan H, Perszyk RE, Zhang J, Kim S, Nocilla KA et al. Classification of missense variants in the N-methyl-d-aspartate receptor GRIN gene family as gain- or loss-of-function. Hum Mol Genet 2023; 32(19):2857–71.

2. Xu Y, Song R, Chen W, Strong K, Shrey D, Gedela S et al. Recurrent seizure-related GRIN1 variant: Molecular mechanism and targeted therapy. Ann Clin Transl Neurol 2021; 8(7):1480–94.

3. Traynelis SF, Burgess MF, Zheng F, Lyuboslavsky P, Powers JL. Control of voltage-independent zinc inhibition of NMDA receptors by the NR1 subunit. J Neurosci 1998; 18(16):6163–75.

4. Swanger SA, Chen W, Wells G, Burger PB, Tankovic A, Bhattacharya S et al. Mechanistic Insight into NMDA Receptor Dysregulation by Rare Variants in the GluN2A and GluN2B Agonist Binding Domains. Am J Hum Genet 2016; 99(6):1261–80.

5. Li J, Zhang J, Tang W, Mizu RK, Kusumoto H, XiangWei W et al. De novo GRIN variants in NMDA receptor M2 channel pore-forming loop are associated with neurological diseases. Hum Mutat 2019; 40(12):2393–413.

6. Ogden KK, Chen W, Swanger SA, McDaniel MJ, Fan LZ, Hu C et al. Molecular Mechanism of Disease-Associated Mutations in the Pre-M1 Helix of NMDA Receptors and Potential Rescue Pharmacology. PLoS Genet 2017; 13(1):e1006536.

7. XiangWei W, Kannan V, Xu Y, Kosobucki GJ, Schulien AJ, Kusumoto H et al. Heterogeneous clinical and functional features of GRIN2D-related developmental and epileptic encephalopathy. Brain 2019; 142(10):3009–27.

8. Xie L, McDaniel MJ, Perszyk RE, Kim S, Cappuccio G, Shapiro KA et al. Functional effects of disease-associated variants reveal that the S1-M1 linker of the NMDA receptor critically controls channel opening. Cell Mol Life Sci 2023; 80(4):110.

9. Yuan H, Hansen KB, Zhang J, Pierson TM, Markello TC, Fajardo KVF et al. Functional analysis of a de novo GRIN2A missense mutation associated with early-onset epileptic encephalopathy. Nat Commun 2014; 5:3251.

10. Chen W, Tankovic A, Burger PB, Kusumoto H, Traynelis SF, Yuan H. Functional Evaluation of a De Novo GRIN2A Mutation Identified in a Patient with Profound Global Developmental Delay and Refractory Epilepsy. Mol Pharmacol 2017; 91(4):317–30.

11. Han W, Yuan H, Allen JP, Kim S, Shaulsky GH, Perszyk RE et al. Opportunities for Precision Treatment of GRIN2A and GRIN2B Gain-of-Function Variants in Triheteromeric N-Methyl-D-Aspartate Receptors. J Pharmacol Exp Ther 2022; 381(1):54–66.

12. Aykul S, Martinez-Hackert E. Determination of half-maximal inhibitory concentration using biosensor-based protein interaction analysis. Anal Biochem 2016; 508:97–103.

13. Yuan H, Erreger K, Dravid SM, Traynelis SF. Conserved structural and functional control of N-methyl-D-aspartate receptor gating by transmembrane domain M3. J Biol Chem 2005; 280(33):29708–16.

14. Traynelis SF. Software-based correction of single compartment series resistance errors. J Neurosci Methods 1998; 86(1):25–34.

15. Perszyk RE, Kristensen AS, Lyuboslavsky P, Traynelis SF. Three-dimensional missense tolerance ratio analysis. Genome Res 2021; 31(8):1447–61.

16. Chou T-H, Tajima N, Romero-Hernandez A, Furukawa H. Structural Basis of Functional Transitions in Mammalian NMDA Receptors. Cell 2020; 182(2):357-371.e13.

17. Chou T-H, Epstein M, Michalski K, Fine E, Biggin PC, Furukawa H. Structural insights into binding of therapeutic channel blockers in NMDA receptors. Nat Struct Mol Biol 2022; 29(6):507–18.

18. Xu Y, Song R, Perszyk RE, Chen W, Kim S, Park KL et al. De novo GRIN variants in M3 helix associated with neurological disorders control channel gating of NMDA receptor. Cell Mol Life Sci 2024; 81(1):153.
